# Experiences, Beliefs, and Dynamics in Chemsex: A Qualitative Meta-Synthesis Among Gay, Bisexual, and Other Men Who Have Sex with Men

**DOI:** 10.3390/healthcare14183071

**Published:** 2026-09-18

**Authors:** Rubén Maldonado Alía, Juan M. Leyva-Moral, David Giménez-Díez, Carolina E. Watson, Nina Granel-Giménez, Rebeca Gómez-Ibáñez, David Téllez-Velasco, M. Dolors Bernabeu-Tamayo

**Affiliations:** 1 Institut Català De La Salut (ICS), 08007 Barcelona, Spain; 2 Department of Nursing, Universitat Autònoma de Barcelona, 08193 Bellaterra, Spain; juanmanuel.leyva@uab.cat (J.M.L.-M.); david.gimenez@uab.cat (D.G.-D.); carolina.watson@uab.cat (C.E.W.); nina.granel@uab.cat (N.G.-G.); rebeca.gomez@uab.cat (R.G.-I.); david.tellez@uab.cat (D.T.-V.); mariadolors.bernabeu@uab.cat (M.D.B.-T.)

**Keywords:** chemsex, gay, bisexual and other men who have sex with men (GBMSM), qualitative meta-synthesis, systematic review, thematic analysis, harm reduction, sexual health

## Abstract

**Background:** Chemsex is described as the intentional use of psychoactive substances to engage in sexual activity over prolonged periods, primarily among gay, bisexual, and other men who have sex with men. Despite its clinical and public health relevance, the available qualitative evidence is scattered and requires interpretive synthesis. **Objective:** To synthesize and interpret the qualitative evidence on chemsex among gay, bisexual, and other men who have sex with men, identifying the main experiential and sociocultural themes and their implications for clinical practice and public health. **Methods:** A qualitative meta-synthesis was conducted following the recommendations of the Preferred Reporting Items for Systematic Reviews and Meta-Analyses (PRISMA) statement. Searches were carried out in PubMed, Dialnet, Scopus, CINAHL, and PubPsych, in Spanish and English, with no time restrictions. Qualitative studies, mixed-methods studies, and reviews reporting qualitative findings in English, Spanish, or Catalan were included, while quantitative, theoretical, or methodologically insufficient studies were excluded. Critical appraisal was conducted using the Critical Appraisal Skills Programme (CASPe) tool. Analysis was performed through inductive thematic analysis. **Results:** A search of electronic databases and grey literature yielded 4973 records. After removing duplicates and screening titles and abstracts, 174 full texts were assessed for eligibility. Following CASPe methodological appraisal, 31 studies met all criteria and were included in the final synthesis. The analysis produced 276 codes and was organized into six interpretive themes: (1) Access to drugs and sex: they are there when I want them; (2) Beliefs and attitudes toward chemsex: internalizing social constructions; (3) Chemsex practice as an identity-based subculture; (4) Patterns of use: of substances, relationships, and spaces; (5) Motivations surrounding chemsex: pleasures and emotions; and (6) Self-perceived risks in chemsex practice: beyond the biological. **Conclusions:** The meta-synthesis portrays chemsex as a sociocultural phenomenon mediated by technology, shared meanings, and relational dynamics, with perceived benefits and potential repercussions. Across studies, a stable tension emerges between psychosocial functions (e.g., pleasure, disinhibition, social connection, and belonging) and cumulative harms that include mental health and relational consequences. The findings support comprehensive and culturally competent interventions that move beyond an exclusively biomedical approach by integrating harm reduction, situational risk assessment, and mental health care, while avoiding moralizing framings that may reduce service acceptability.

## 1. Introduction

Change processes and problems that defy simple solutions are not uncommon in the sphere of healthcare. Some of them involve insufficient therapeutics, entanglement of psychosocial, cultural, and relational factors, or lack of empirical evidence. The problem of chemsex represents a case in point, becoming increasingly relevant in terms of public health implications in the course of continuous evolution [1]. Indeed, it is a heterogeneous and fairly novel phenomenon that poses certain difficulties for both conceptualization and clinical management [1].

In general, the phenomenon is understood as purposeful consumption of psychoactive substances that facilitate sexual activity over extended periods of time among gay, bisexual, and other men who have sex with men (GBMSM) [2]. Chemsex has attracted much attention in terms of public health due to its associations with sexually transmitted diseases, risks of HIV transmission, potential medical problems, mental issues, as well as complicated psychosocial and cultural dynamics [3]. However, besides the above-mentioned aspects, chemsex has also acquired qualitative characteristics, becoming a phenomenon based on shared scripts and expectations associated with pleasure, heightened sensations, disinhibition, and particular forms of intimacy [3]. This double aspect becomes especially problematic since chemsex may be a form of satisfaction but also a convergence point for various risk activities that exacerbate preexisting vulnerabilities or re-activate certain traumatic experiences.

The phenomenon appears to be more prevalent in the urban environment, which implies fundamental changes in partner selection facilitated by digital media and the dynamics of organizing sexual encounters. In such a situation, chemsex emerges as a subculture practice based on the principles of collectivity and specific shared expectations and regulations regarding practices, risk behaviors, and boundaries [4]. Notwithstanding the importance of the psychological, relational, and cultural aspects of the phenomenon, little attention has been paid to them in the existing literature.

In addition, the fear of being morally judged, stigmatized, and misunderstood by clinicians constitutes an important barrier to seeking professional help for people involved in chemsex [5]. Given that the perception of chemsex may imply certain stigma and risks of being disclosed, management of information, silence, and avoidance emerge as potentially adaptive behavioral strategies, although these do not contribute to prevention and reduction of harm. Here, HIV literature can provide useful insights, as specific processes contributing to stigma, such as anticipated rejection, stereotyping, and internalization, have been associated with negative psychological outcomes and reduced utilization of health services [6]. All this highlights the need for theory-driven and evidence-based concepts aimed at expanding the competence of clinicians and facilitating effective intervention.

Indeed, harm reduction approaches and culturally adapted intervention programs appear to be more relevant in managing chemsex than general drug treatments that cannot fully account for sexuality, digital sociality, polysubstance use, long sessions, and risky behavior [3,4]. What the literature repeatedly emphasizes is the significance of understanding what the practice means to participants and what needs it fulfills for them. Understanding how the process is regulated and what kind of perceptions and risks exist within the community is equally important.

However, notwithstanding the substantial growth of research outputs over recent years, qualitative evidence related to chemsex remains scattered among different fields and developed on the basis of noncommensurable theories. Thus, synthesizing and integrating evidence into clinical guidelines poses particular challenges and contributes to a silo approach to the problem, according to which sexual health, mental disorders, and substance abuse remain distinct areas. Conducting a metasynthesis is likely to allow moving away from these limitations and integrating available knowledge in order to generate a unified picture of chemsex. While previous reviews have mapped the epidemiological or clinical outcomes of chemsex, this metasynthesis adds to the existing literature by strictly focusing on the experiential, social, and relational themes, integrating qualitative evidence to highlight the structural ambivalence of the practice.

Therefore, what is required in order to generate a more coherent and clinically valuable understanding of chemsex is systematic re-interpretation of available evidence that focuses on meanings, social dynamics, and sociocultural context. This involves understanding how beliefs about chemsex become socially constructed and how the materiality of chemsex is organized across various settings. In addition, the ambivalence that keeps the practice in place must be taken into consideration in the development of clinical and community interventions [3,4]. The objective of the current research is to conduct a qualitative meta-synthesis of existing evidence on chemsex among GBMSM and integrate the results into interpretative themes.

## 2. Materials and Methods

A qualitative meta-synthesis of the available literature on chemsex among gay, bisexual and other men who have sex with men (GBMSM) was conducted. The aim was to integrate and interpret prior qualitative findings in order to develop a thematic understanding of the phenomenon, beyond the mere aggregation of results. The planning and reporting of this qualitative evidence synthesis followed the ENTREQ statement [7] and PRISMA 2020 recommendations [8]. Completed checklists are provided as Supplementary Tables S1 and S2.

The protocol for this qualitative metasynthesis was registered in PROSPERO under the registration number CRD42023434687 (Record ID/CRD Accession Number: 434687). A systematic search was conducted between October 2022 and January 2023 in PubMed, Scopus, CINAHL, and PubPsych, as well as in Dialnet to broaden the capture of Spanish-language literature. The search strategy was designed to capture relevant qualitative literature across disciplines, including sociological research, provided it met the inclusion criteria. It combined Boolean combinations of terms related to the phenomenon (chemsex, sexualized drug use, sexualised substance use) and to the population of interest (MSM, gay, bisexual, GBMSM, etc.), adapting syntax and subject headings to each database (see example in Table 1). The Boolean operator AND was used in English, alongside the conjunction “y” in Spanish. Searches were conducted in English, Catalan, and Spanish, with no time restrictions, to avoid date-related bias given the relative novelty of the term and the possibility of earlier studies using different nomenclature. In addition, grey literature was purposively reviewed, such as technical documents and reports, and incorporated when it contained relevant and verifiable qualitative results. Indeed, Google Scholar was searched to identify grey literature, using combinations of the terms “chemsex”, “sexualized drug use”, “qualitative”, “interviews”, “thesis”, and “report”. The first 100 results ranked by relevance were screened.

Study selection was performed by three pairs of reviewers and a pair of independent judges. Inclusion criteria encompassed qualitative studies, mixed methods studies, metasyntheses, and systematic reviews providing interpretive qualitative findings on chemsex or sexualized substance use among gay, bisexual, and other men who have sex with men, published in English, Spanish, or Catalan. We excluded studies involving individuals under 18 years of age, research conducted outside the population of interest, exclusively quantitative studies without a qualitative component, and theoretical papers or essays without an empirical basis. Furthermore, mixed methods articles, systematic reviews, or meta-analyses where qualitative results could not be separated from quantitative outcomes were excluded. To prevent double counting, when reviews reporting qualitative findings were eligible, we strictly extracted only the primary qualitative data explicitly attributed to the original studies, thereby maintaining a clear distinction between primary evidence and secondary interpretation.

**Figure 1:**
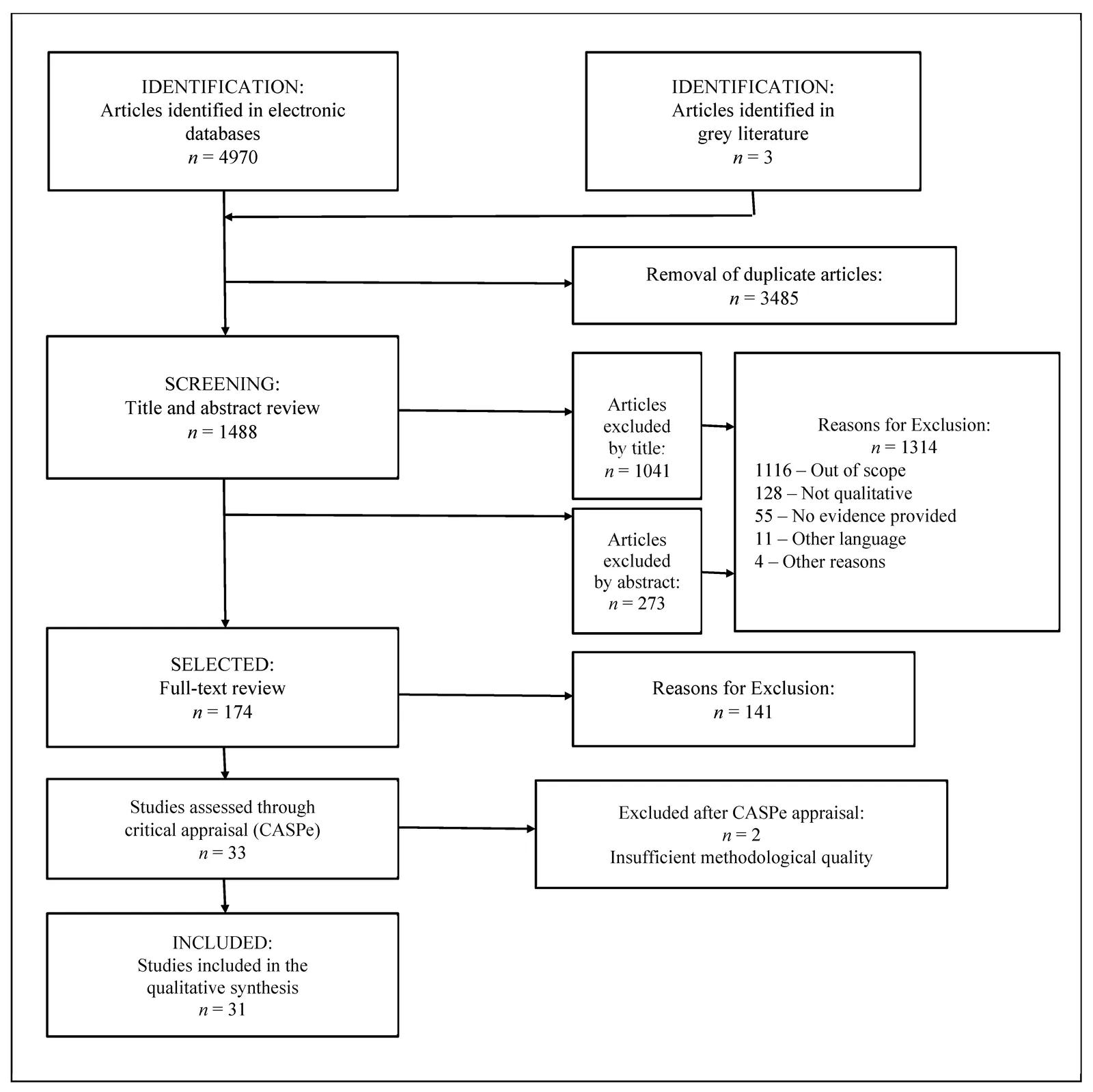
PRISMA Flow.

The 33 studies that met the eligibility criteria were critically appraised using the CASPe qualitative research checklist. The appraisal was used to establish a minimum threshold of methodological rigor for inclusion rather than to assign numerical weights to the findings. Exclusion was based on an overall reasoned assessment across the CASPe domains rather than on a single item or numerical cut-off. The remaining 31 studies were retained for the synthesis. Detailed quality-appraisal results for each included study are provided in the Supplementary Table S3. The selection process was conducted in successive phases. A total of 1488 records were screened by title, of which 447 proceeded to abstract screening. After 273 records were excluded at the abstract stage, 174 full-text reports were assessed for eligibility. Of these, 141 were excluded before critical appraisal, leaving 33 studies for CASPe assessment. Two studies were subsequently excluded because of insufficient methodological quality, resulting in a final corpus of 31 studies. Reasons for exclusion were systematically recorded throughout the process to ensure transparency and traceability (Figure 1).

Following the search, the CASPe tool was utilized to establish a minimum threshold of methodological rigor for inclusion, rather than to assign numerical weights to the findings. Studies that showed methodological limitations considered incompatible with a qualitative metasynthesis, according to CASPe quality criteria, were excluded [9]. This appraisal was used as a reasoned judgement of rigor to ensure that the synthesis was built on qualitative evidence with a minimum degree of methodological consistency.

The selection process was conducted in phases. First, titles and abstracts were screened to identify potentially relevant studies; subsequently, full texts were assessed to confirm eligibility. In this final phase, reasons for exclusion were systematically recorded so that the process would remain traceable and replicable. As this was a synthesis of published studies, the work did not involve intervention with participants nor the collection of primary data; nevertheless, a transparent methodological logic was prioritized to avoid selection bias and to clearly justify how the final corpus of articles was derived.

The synthesis was conducted using inductive thematic analysis, conceptualizing analysis as a progressive process of coding, organization, and thematic refinement [10]. Consistent with the logic of thematic syntheses in qualitative reviews, initial coding generated descriptive themes that were subsequently grouped and reinterpreted to construct higher-order analytical themes, seeking to integrate convergences and tensions across studies [11]. At this stage, the aim was to sustain an interpretive structure capable of capturing the phenomenon in its complexity, while respecting contextual differences and nuances. Coding was conducted by RMA (he/his) using the qualitative data analysis software ATLAS.ti (V23), under the supervision of MDBT (she/her) and JMLM (he/his). Disagreements that arose during the analytical process were resolved through critical and constructive discussions among the research team. Subsequently, codes were collapsed into broader themes based on content similarities or theoretical arguments. Interpretive saturation was considered to be achieved when the integration of data from additional studies no longer generated novel codes or altered the fundamental interpretive structure of the developed themes. To confirm the final findings, a concluding discussion meeting was held with experts in the topic and the methodology, who suggested no changes to the final analytical version. All direct quotations presented in the results are verbatim excerpts from the primary articles. For the single included study published in Spanish, data extraction and initial coding were conducted in the original language. Once the final interpretive findings were established, the selected quotations were translated into English and subsequently back-translated by two bilingual researchers to guarantee semantic accuracy and prevent the loss of original meaning.

## 3. Results

Following the search, screening, and critical appraisal process, 31 studies were included in the final synthesis (see Table 2). The 31 included studies were published between 2002 and 2022 and were conducted predominantly in urban settings. Across the corpus, European and Anglophone contexts were most frequent, with multiple studies based in the United Kingdom (notably London), alongside other European sites (e.g., Sweden/Germany, Spain, Portugal, Italy, Ireland) and additional studies from Canada, Australia, and Asia (particularly Singapore and Malaysia). Methodologically, most studies relied on qualitative designs based on semi-structured interviews, sometimes complemented by focus groups or other qualitative materials. In terms of analysis, inductive thematic analysis was the most commonly reported approach, while a smaller number of studies employed discourse-analytic, phenomenological/interpretative, narrative, or ethnographic/autoethnographic strategies. An inductive coding process based on the qualitative findings of these works generated 276 codes, which were subsequently reorganized into six interpretive themes.

To illustrate the distribution of evidence and clarify which contexts are represented, a matrix detailing the specific studies that contributed to each of the six interpretive themes is provided in Table 3.

Overall, the data indicate that chemsex, beyond substance use itself, is mediated by multiple interrelated factors: accessibility (to drugs, sex, and technology), the construction of meanings and shared expectations, specific subcultural and identity-related logics, key psychological and relational functions (pleasure, emotional regulation, coping), all coexisting with risks, harm management strategies, and barriers to accessing healthcare resources. The findings were structured around the following themes:

### 3.1. Access to Drugs and Sex: It Is There When I Want It

The underlying notion within this theme entails the comparative ease of acquiring drugs or sexual partners, or both, in relation to chemsex activities and their social contexts, which can include mobile apps, nightlife, LGBTQIA+ venues, and similar places. The core of this theme lies in the assumption that the availability of either drugs or sex (or both) does not simply provide the social environment for people to take part in chemsex but becomes an actively operating element influencing their choices about participating in such activities and the amount of their participation. This type of accessibility varies based on location, regulatory practices, historical and cultural context, openness toward sexuality, and many other factors.

At the same time, this accessibility is perceived as a feature of immediacy by those who participate in chemsex rather than as a technical or infrastructural aspect of this phenomenon, which is why it may be described as being ubiquitous:

“*It’s just everywhere. (…). I mean, I can’t think of any place where I haven’t seen drugs being used*”.

[12]

The data suggest that chemsex operates as a type of ecosystem in which multiple forms of access intersect: access to drugs, sex, networks, and spaces. For example, some accounts describe the ease of obtaining what one seeks directly through applications: “*Ease of access is a big problem. You can really get whatever you want through Grindr”* [3]. Technological accessibility emerges as a structuring component: apps reorganize availability, timing, and expectations, and they introduce specific codes that render the phenomenon visible within the platform itself. They also introduce codes that make the phenomenon visible within the platform itself: *“If you go on Grindr, there are many profiles that state ‘CF’… another way of saying: do you want to have fun with drugs?”* [38].

In parallel, some accounts describe how such hyperavailability may foster addictive patterns toward the technology itself, particularly when combined with prolonged sessions and sleep deprivation: “*Something horrible happens when people just sit there awake for days, taking drugs and looking for connections on Grindr on their phones. All the time, ‘look at this guy, look at this guy’… It’s like a monkey brain in a slot machine*” [39]. For some participants, drugs are not merely substances but tools that facilitate entry into groups, socialization, and the experience of belonging. One testimony explains this clearly: “*If you approach someone who is doing chemsex, you can be sure that person is open to having sex with someone else who is also using drugs… the drug part is just a tool… and you’re perceived as part of the community*” [24].

### 3.2. Beliefs and Attitudes Toward Chemsex: Internalizing Social Constructions

This theme refers to how individuals internalize and adopt various social perceptions and attitudes related to chemsex. It derives from broader perceptions surrounding chemsex and drug use and encompasses a range of codes addressing different aspects of the phenomenon. These include the popularity and normalization of chemsex, the minimization or normalization of drug use, positive and negative perceptions of chemsex, perceptions of drugs, perceptions of sex and casual sex, as well as lived experiences and myths surrounding chemsex practices, among others.

A salient element emerging from the data is the social dimension of desire: not participating in chemsex may be experienced as being excluded from something that, in certain contexts, is framed as exciting or almost necessary to “belong.” One testimony expresses this directly: “*Almost everyone talks about chemsex in the gay community as an extremely exciting activity… not participating… makes you feel a bit excluded*” [27]. In certain circuits, chemsex may come to be perceived as expected or normalized, and this expectation can be internalized relatively quickly, particularly when intertwined with a desire for connection, curiosity, and the belief that “everyone is doing it” [27]. At the same time, such normalization does not necessarily imply the absence of conflict; rather, it is often structured as ambivalence. On the one hand, chemsex is constructed as exciting and desirable; on the other, participants remain aware that it may entail negative consequences. Testimonies reflecting minimization or rationalization of practices are also frequent: “*I tell myself: it’s fine… I’m still doing my job, and I’ve only been sick three times this month*” [12].

In this regard, the data suggest that chemsex involves processes of cognitive and emotional adjustment that enable the continuation of the practice, particularly when it is perceived as functional or rewarding. Experiences of chemsex may be lived as simultaneously positive and negative, and several testimonies indicate that these experiences fluctuate depending on context, emotional state, type of session, and what occurs afterward. Additionally, the data reveal participants’ perceptions of sex, drugs, and chemsex more broadly. Some testimonies illustrate how beliefs about sexual intensity and bodily experiences are constructed and how these perceptions may influence behavior. In this sense, one participant noted: “*You want to experience more sensations… you think not using a condom will make you feel more euphoric… when they use a condom, they don’t feel the same intensity*” [38].

### 3.3. The Practice of Chemsex as an Identity Subculture

This theme refers to aspects related to gender identity and sexual orientation, as well as to the culture, values, and social practices of the LGBTQ+ community, including the use of specific symbols, language, and narratives that form part of chemsex subculture. It encompasses participants’ perceptions of themselves as belonging to such a subcommunity, the acceptance of sexual diversity, gender norms and roles, and the influence of popular culture and history in shaping identity and subjectivity. Chemsex is described as a social or cultural reality, as illustrated in the following excerpt: “*It’s a kind of rite of aggregation for gay men involving sex and drugs. It has specific codes and scripts. There’s also an expert guide you can trust and let yourself go with*” [26].

These attributions and constructions appear to be shaped by the dominant sociocultural and political paradigm, as narratives frequently frame consumerism, liberalism, hedonism, casual sex, and promiscuity as central elements of masculinity. Such constructions of masculinity include an ideal model—also historically and socioculturally determined—often characterized by the rejection of emotional expression or sensitivity, the rejection of effeminacy (stereotypically feminized gender expression) and passive sexual roles, alongside the exaltation of sexual potency, performance, and a well-shaped muscular appearance. Some testimonies reflect this critical view of the broader gay scene: “*The gay scene is bad wherever you go. In London, it’s very critical because on apps, if you don’t have defined abs, you’re considered fat. If you’re not the alpha male, you’re effeminate. It’s hard to see how anyone can have self-esteem like that. In fact, it makes you feel unattractive and unwanted… None of that exists in chemsex…*” [21]. Other accounts emphasize a consumerist logic underlying sexual encounters: “*Chemsex is like eating at an all-you-can-eat buffet… Now you can buy sex in the supermarket and it doesn’t last long—it’s consumerist sex*” [27].

In addition, the data include numerous narratives highlighting traumatic experiences and/or experiences of rejection among GBMSM who engage in chemsex—whether occurring within or outside chemsex contexts, and within or outside the LGBTQ+ community—as well as experiences of rejection and/or discrimination related to living with HIV. “*Coming out to my parents was horrible. In the end they accepted me, but the memorable part—the part you always remember and that stays with you—is that it was horrible. My mother cried in front of me, and even though they accepted and understood it, you just know that (deep down) it’s something they wish wasn’t so. I still think to myself: ‘she was never completely happy about it and she never will be.’*” [21].

### 3.4. Patterns of Use: Substances, Relationships, and Spaces

This theme focuses on the characteristics of chemsex itself, detailing, for example, which substances are used, where they are consumed, and the contexts in which chemsex takes place, among other aspects. It therefore includes analysis of the different drugs used in chemsex contexts, modes of administration, dosage and frequency of use, patterns of consumption, contexts of use, polysubstance use, and all elements related to various psychoactive substances, as well as their physiological and psychological effects. Narratives related to this category are among the most frequent, and the following example is provided as representative: “*From my point of view, in chemsex, G (GHB) and meph (mephedrone) are mainly used together; and there are those who take both, but consume large amounts of Tina (methamphetamine). (Although in reality) I think that when you’re in chemsex, they ask you if you use Tina, and (if so) you’ll be in one group (of people); if you use meph, you’ll be in another group*” [12].

Accounts from participants frequently point to a close relationship between patterns of substance use and the pursuit of specific pharmacological effects—whether produced by individual substances or by particular combinations thereof. This selectivity suggests that substance choice within chemsex contexts is rarely arbitrary, but rather guided by the effects users seek to achieve in a given encounter. When disinhibition or psychological detachment is the primary aim, central nervous system depressants such as GHB tend to predominate. By contrast, when the desired outcome involves heightened sociability or psychostimulant effects, methamphetamine emerges more commonly as the substance of choice. Among the most consistently reported motivations is the enhancement of sexual performance: substances are employed as a means of sustaining or improving one’s capacity to engage sexually, effectively functioning as resources that supply the user with increased energy, endurance, and a reduced sense of physical limitation. For instance, one testimony states: “*So for me and this guy, we smoked crystal together and I literally fucked him for 12 h non-stop, and it was incredible. You feel superhuman; he can go on and on. You just keep giving and giving*” [40].

Accounts also commonly highlight the emergence of mutual support networks and peer care within chemsex contexts, as illustrated by the following testimony: “*I understand the patterns of the people I usually practice chemsex with. I know when they’ve taken too much GHB. My friends are my community. I keep their keys. I make sure there’s enough water. I send emails reminding them to take their medications. If I have to play that role, it’s fine, because I’d rather my friends be safe and secure, meet them the next day, and know they’re okay*” [35].

### 3.5. Motivations Surrounding Chemsex: Emotions and Pleasures

This theme explores the motivations and/or expectations that lead GBMSM to engage in chemsex, focusing on the underlying reasons for this practice. It encompasses motivations related to seeking new or intensified emotions, sensations, and experiences, as well as motivations aimed at avoiding unpleasant thoughts, emotions, and memories. It also includes social, group-based, and individual motivations. Furthermore, chemsex may function as a coping strategy for managing diverse life experiences, addressing events perceived as difficult, or serving as a key resource to enhance sexual performance and overall sexual experience.

The following account illustrates several motivations associated with engaging in chemsex, including accessing sexual partners, seeking intense experiences, attempting to relive the first session, or experimenting with substances: “*My first time was one day when I was chatting with a guy on Grindr and I went to his house, but when I arrived, there wasn’t just one guy, there were fifteen. At first, I was a bit scared. But it was like: lots of people, pornography on the TV, great music, people were naked… and it was so attractive… so I stayed. And I really liked the guys, I liked the atmosphere, people looked really cool… and then I realized there were drugs, but I didn’t mind and I stayed, and after a while I wanted to try them too. And it was great. I have incredible memories of it. For me, I don’t know how it is for others, but group sex had always been a fantasy. And these guys were so handsome, so hot, everyone was naked, the vibes were incredible. Everything was perfect. And when I got home, it was like: I had spent the whole weekend in an incredible house, with amazing guys, using drugs, I had the best time of my life. It was perfect. Perfect. And that’s the bad thing: that’s what makes you want to do it again. But it’s impossible to recreate*” [31].

Therefore, motivations for initiating and maintaining chemsex extend beyond substance use itself, emphasizing emotional, psychological, relational, and belonging-related components as key elements. For example, the following testimony links chemsex with psychological factors: “*For me, it’s been something really positive (participating in chemsex), like it’s helped my confidence enormously… I’ve made lots of friends at chemsex parties and that (…)*” [20].

### 3.6. Self-Perceived Risks in the Practice of Chemsex: Beyond the Biological

This theme addresses the risks, consequences, and difficulties associated with substance use and participation in chemsex, as well as the harm reduction and risk management strategies that GBMSM draw upon in attempting to mitigate the impact of these practices on their lives. Its scope is broad, encompassing perceived risks linked to chemsex engagement, the occupational and social disruption that problematic substance use may produce, the negative sequelae of consumption, overdose risk, levels of awareness around use, dose self-regulation, and the particular difficulties individuals encounter when attempting to discontinue their involvement in chemsex.

Beyond these more immediate dimensions, the theme also attends to the role of expert guidance, the pursuit of balance between use and everyday functioning, the maintenance of occupational and social routines, mutual support among peers, the erosion or absence of boundaries within chemsex contexts, and the ambivalence that frequently characterizes individuals’ relationships with their own consumption. Harm reduction strategies operative at both the substance use and the chemsex levels are examined alongside the need for accessible, accurate information and for professional services specifically oriented toward this population. The theme further extends to encompass the grief and loss processes that may accompany disengagement from chemsex, the active avoidance of cessation, the need for sustained healthcare support, and the broader psychosocial implications of prolonged involvement. Mental health difficulties associated with chemsex, patterns of excessive consumption, recovery trajectories, challenges in establishing personal limits, and risk behaviors are also considered. Finally, the theme foregrounds the need for structured education, community-based organizational responses, clear frameworks around consent and responsible use, and equitable access to risk management information.

Across multiple accounts, participants emphasize that perceived implications and risks extend beyond purely biological dimensions, as illustrated in the following example: “*[Chemsex] is like a ticket to paradise in five minutes. You can’t reach such a euphoric state and have so much fun in your everyday life (…) You can hardly achieve it any other way than by using drugs. But it comes at a cost. (…) The beauty of many other things fades away. Why would you stay home on a Saturday having dinner with your family when you could be dancing in paradise and feeling extremely good? Having so much fun can be dangerous, because everything else becomes much more boring*” [14].

Numerous narratives also describe how participants actively protect their health within chemsex settings. For example: “*[Chemsex] has specific codes and scripts. These (codes) constitute a kind of protection for our health. For example, we take notes together when we use GHB, writing it down on a table. There is also the presence of a trusted person, an experienced guide you can rely on so that you can let yourself go*” [26].

Data are also abundant regarding the consequences of chemsex sessions. The effects and repercussions may range from the need to recover for a short period following a session to more profound and lasting changes in the individual. Not all individuals who participate in chemsex experience the same effects, nor to the same degree, and such effects do not necessarily entail a significant loss of functioning or severe harm in every case. The following account illustrates the impact reported by some GBMSM: “*Two years ago, for a period of time, I became really addicted to Tina (methamphetamine). And it affected my work, my studies, and my life very negatively, because I used to take drugs almost every day. I would go out and not come back home. My parents were worried. I disappeared for three days without telling my family anything. And I kept missing classes. I didn’t hand in my assignments. The teachers got angry with me, my supervisor got angry with me, and in the end I lost everything. I had to drop out of my studies because I missed too many classes and couldn’t take the exams*” [38].

## 4. Discussion

The findings of this metasynthesis point toward chemsex as a fundamentally relational and situated phenomenon, one in which substance use is embedded within a sexual script shaped by norms, expectations, motivations, lived experiences, perceptions, and accessibility. The intensification of pleasure, disinhibition, prolonged sexual encounters, attenuation of social anxiety, and facilitation of interpersonal contact are consistently identified as central to the practice; yet these same features may simultaneously heighten exposure to biological, psychological, and social harm [4,42]. This coexistence between the psychosocial functions that chemsex serves and the costs it may entail cannot be adequately reduced to an individual-level problem: many of the motivations reported across studies are described as socially shared and rooted in the pursuit of recognition and belonging—that is, in a need to be part of a community with which one identifies. Consequently, the themes resulting from this metasynthesis do not merely aggregate qualitative findings, but they actively interrogate and contrast this fundamental tension between the psychosocial functions of chemsex and its cumulative harms.

A cross-cutting element throughout the corpus is digital mediation as an organizing condition of the phenomenon rather than a mere facilitative backdrop. Wang et al. [43] describe shared norms and repertoires that render chemsex both attractive and socially intelligible for certain gay men and other MSM, and frame technological accessibility—encompassing contact, coordination, and availability—as a mechanism that reduces friction and supports the continuity of sessions. Technology does not, in itself, explain chemsex; rather, it reorganizes encounters in terms of temporality and opportunity, fostering maintenance dynamics that, in certain cases, acquire clinical salience [43,44]. From this standpoint, interventions focused narrowly on the transmission of risk information are likely to prove insufficient if they fail to account for the situational mechanisms that sustain the practice and impede the reintroduction of deliberative agency in contexts characterized by immediacy, high levels of stimulation, and prolonged sequential engagement. However, these interpretations regarding subcultures and relational ecologies must be applied cautiously. They primarily reflect the experiences of individuals within specific urban, European, and Anglophone settings and may not apply equally across all contexts, age groups, HIV statuses, or patterns of substance use. Consequently, these findings should not be utilized to essentialize the broader community of gay, bisexual, and other men who have sex with men, recognizing that engagement in chemsex is highly variable and context-dependent.

Our synthesized findings suggest that, in certain contexts, chemsex operates as a practice of socialization and communal belonging, a dynamic that is increasingly supported by recent external literature [42,45]. Chemsex is, therefore, more than a pattern of substance use: it is a psychological, social, and relational phenomenon in its own right. At the same time, the literature indicates that chemsex may reproduce social hierarchies, gendered roles, and normative demands—including expectations around sexual availability and performance, body aesthetics, and social status, among others—which helps to account for why the practice can be experienced as simultaneously inclusive and coercive, and why the distress that sometimes follows cannot be reduced to a pharmacological comedown or its physical consequences alone [45,46].

What this metasynthesis ultimately points to is not a dichotomy between community and risk, but rather their sustained coexistence within chemsex—and it is precisely this coexistence that structures the ambivalence that surfaces repeatedly across participants’ accounts. Ambivalence, in this context, deserves greater conceptual precision than it is typically afforded. It is not a superficial oscillation between enjoyment and regret, but a stable and enduring coexistence of positive reinforcers and tangible costs that may persist over years. The result is a relatively stable functional conflict: chemsex supplies immediate and socially meaningful reinforcers—pleasure, disinhibition, access to contact, a sense of belonging—while its costs tend to be cumulative or temporally displaced, manifesting as affective depletion, guilt, shame, relational difficulties, and a growing sense of diminished agency [42,44,47]. This pattern carries direct clinical relevance, as it suggests that behavioral change does not hinge primarily on increasing risk awareness, but on developing an understanding of the functions chemsex serves in an individual’s life and on identifying viable alternatives capable of meeting those functions without equivalent exposure to harm.

Consistent with our metasynthesis, the treatment of risk in recent external qualitative literature also reflects a meaningful departure from a strictly biomedical framework. Studies show concerns about HIV, sexually transmitted infections, or the pharmacological effects of substances; they extend to subjective and social consequences that receive comparatively less clinical attention, including post-session anxiety, dysphoria, shame, social isolation, breaches of relational trust, and an increasingly conditioned relationship with one’s own desire [46,48]. This broader conception of harm carries direct implications for service design: a provision model organized around sexual health alone, or around substance use in isolation, is unlikely to address the forms of distress most salient to a significant subset of users. From the perspective of service providers, this challenge is described in terms of responding to clinical presentations in which mental health, sexuality, substance use, and stigma are tightly interwoven—compounded further by structural barriers related to service fragmentation and the low acceptability of provision perceived as moralizing or culturally ill-adapted, a challenge similarly documented in broader chemsex research [49].

A recurring and analytically significant finding across studies is that chemsex settings are not devoid of agency or care practices. Tacit norms and informal harm reduction strategies are described throughout the literature, encompassing accompaniment, mutual monitoring, negotiation of boundaries, and various practices oriented toward minimizing harm without requiring exit from the setting itself [44,47]. Acknowledging these practices does not amount to an idealization of chemsex; rather, it means identifying potential entry points for more credible and co-produced interventions—ones that build upon existing community repertoires rather than operating in opposition to them. This perspective is particularly pertinent given the pronounced heterogeneity of the phenomenon: neither the degree to which chemsex is experienced as problematic nor the trajectories individuals follow are uniform across contexts. Research conducted in Australia, for instance, documents considerable diversity in settings, relationship configurations, and practices, challenging any tendency to construct chemsex as a homogeneous object of intervention [50]; similarly, work carried out in Madrid identifies distinct session typologies that point to meaningfully differentiated modalities of engagement [51]. This diversity demands that prevention and care be conceived not as a single, standardized package, but as a set of differentiated care pathways with staged goals calibrated to individual profiles, phases of involvement, and specific contexts.

From a clinical standpoint, the synthesis supports an integrative approach that combines harm reduction, situational risk assessment, and psychotherapeutic work oriented toward the functions that chemsex serves and the ambivalence that surrounds it. In operational terms, this involves reconstructing the sequences preceding, during, and following sessions; identifying the conditions under which use tends to escalate; attending to the role of digital technology and social networks in sustaining the practice; and working explicitly on the mechanisms through which the chemsex setting meets affective or identity-related needs [42,47]. The evidence also indicates that goal-setting should remain sensitive to individual trajectories. For some, a realistic and clinically appropriate aim will be to reduce frequency or introduce situational modifications; for others—particularly where significant functional impairment, psychiatric comorbidity, or persistent loss of control is present—an abstinence-oriented plan supported by more intensive treatment may be warranted. In either case, what the evidence consistently underscores is that the service model should not place individuals in the position of having to choose between their health and their sense of belonging, but should instead offer meaningful alternatives for connection, sexuality, and emotional regulation that are compatible with both care and autonomy [44,49,52,53,54].

### Limitations

The present metasynthesis is inevitably shaped by the composition of the available literature and by the conditions under which qualitative evidence on chemsex tends to be produced. Most included studies originate from urban settings in high-income countries, with a marked geographical concentration in European and Anglophone contexts. This regional focus limits the transferability of our findings, as geographic differences significantly shape the chemsex subculture. Furthermore, these studies typically recruit participants through sexual health services, community programs, digital networks, or circuits directly connected to chemsex practice. This recruitment pattern may constrain the applicability of certain findings to other sociocultural, healthcare, and legal contexts, specifically environments in which digital infrastructures, resource availability, regulatory frameworks, and the degree of community visibility differ considerably from those represented in the literature. It is equally plausible that the existing body of data disproportionately captures trajectories that are more service-connected or more visible within particular subcultural environments, while leaving underrepresented those experiences that are less accessible to research, including more concealed profiles, individuals with weaker community ties, those facing greater socioeconomic precariousness, or those with more limited access to care. The results of this synthesis should therefore be read as an account of meanings and dynamics documented across a set of contexts with relatively specific and not universally generalizable characteristics.

A further set of limitations stems from the conceptual and methodological heterogeneity that pervades the literature, which in turn constrains comparability across studies. There is no consensual operational definition of chemsex: studies diverge with respect to the substances considered, the practices included, the thresholds of intensity or frequency applied, and the criteria used to delimit sessions and contexts. The analytical frameworks employed—ranging from thematic analysis and phenomenological approaches to critical and syndemic perspectives, among others—also differ in the kinds of questions they make legible, and consequently in the findings they generate. The synthesized data derive predominantly from retrospective self-report in a field heavily permeated by stigma and by moral norms surrounding sexuality, substance use, and HIV/STIs; these conditions may modulate what participants choose to narrate, the level of detail they offer, and the emphases they adopt—for instance, foregrounding dimensions of risk, guilt, or loss of control in ways that may reflect the interview context as much as lived experience. Finally, as is inherent to any qualitative metasynthesis, the identification of codes, their thematic aggregation, and the process of interpretive integration are dependent on analytical decisions that cannot be fully standardized. Even when systematic procedures are applied, an element of interpretive judgement remains unavoidable, and the uneven level of detail reported across primary studies may influence the relative prominence assigned to certain themes while leaving other aspects of the data less fully elaborated.

## 5. Conclusions

The experience of chemsex constitutes a practice that emerges at the intersection of technology, sexuality, subculture, emotional regulation, relational bonds, and the need for belonging. In this sense, what the synthesis ultimately reveals is that what may initially appear as an individual choice—the use of substances to intensify sexual experience—unfolds in practice as a relational ecology in which accessibility, shared meanings, and community dynamics jointly shape both entry into the practice and its continuation over time.

Within chemsex contexts, the subjective experience of accessibility is consistently described in terms of immediacy and ubiquity. This pervasive sense of constant availability does not merely facilitate engagement; it may also foster forms of compulsivity organized around seeking, connecting, and remaining within digital circuits. The synthesis indicates that chemsex becomes particularly likely when access operates as a perceived promise of rapid resolution of needs—for sex, affection, excitement, or belonging—within a framework in which, as participants themselves describe it, everything is there when I want it.

Yet normalization and ambivalence coexist without apparent resolution. The same narratives that describe excitement, disinhibition, and connection also give account of emotional costs, exhaustion, and loss of control. The continuity of the practice is thus sustained not by the absence of awareness, but through cognitive and affective adjustments that allow individuals to integrate—or indefinitely postpone—the recognition of adverse consequences. In certain contexts, as the metasynthesis makes evident, non-participation may itself be experienced as exclusion from a desirable reference group, transforming belonging into a subtle yet persistent mechanism of both attraction and pressure.

Conceptualizing chemsex as an identity-based subculture helps to account for why the practice is organized around specific codes, language, and rituals that extend well beyond substance use per se. The synthesized evidence describes the circulation of shared scripts, the presence of informal guides with experiential authority, and a normative repertoire within which roles, limits, and social status are actively negotiated. Significant tensions surface within this structure: on one hand, chemsex may function as a space in which forms of judgment—related to age, body image, HIV status, stigma, or perceived fit within gay community contexts—are attenuated, and where recognition difficult to obtain elsewhere becomes accessible; on the other hand, within these same circuits, hierarchies, expectations of sexual performativity, and power dynamics may reproduce or amplify pre-existing vulnerabilities rather than dissolve them.

The motivations associated with chemsex constitute a broad and internally varied repertoire that integrates pleasure, exploration, and sensory and sexual intensification alongside coping with distress, emotional avoidance, and the pursuit of relational connection. For some participants, the synthesis suggests, chemsex fulfills psychological and relational functions that are not adequately met in other life contexts: it facilitates intimacy, reduces inhibitions, renders encounters viable, and enables experiences of belonging that may be otherwise difficult to access. This functional dimension—which neither denies the presence of harm nor minimizes its significance—helps to explain why the practice persists even when its adverse consequences are consciously acknowledged, and why clinical and public health approaches anchored exclusively within a biomedical framework are likely to prove insufficient.

Self-perceived risks, as narrated across studies, extend well beyond biological dimensions: experiences of harm encompass effects on mental health, identity, relational life, self-esteem, and the capacity for self-care. It is at precisely this point that a central organizing logic becomes visible—chemsex operates as a field of ongoing negotiation between gratification and cost, connection and depletion, belonging and loss. Ambivalence thus emerges not as an incidental or secondary feature of the phenomenon, but as one of its defining structural characteristics.

Taken together, the metasynthesis suggests that any clinical or preventive response with genuine aspirations to effectiveness must recognize this complexity, integrate the cultural and community dimensions of the practice, and sustain non-pathologizing, culturally competent approaches. Rather than reducing chemsex to an expression of individual deviation or failure of self-regulation, it warrants understanding as a relational experience mediated by inequalities, stigma, and deeply felt needs for recognition.

From a clinical standpoint, the findings support the value of an integrative approach that combines harm reduction, situational risk assessment, and psychotherapeutic work explicitly oriented toward ambivalence—understood here not as therapeutic resistance, but as a structural feature of the phenomenon that requires direct engagement. In practice, this entails maintaining a therapeutic space in which individuals are able to articulate both the benefits and the costs of chemsex simultaneously, without being positioned defensively, and in which the specific mechanisms through which technological accessibility, subcultural scripts, and relational pressures contribute to escalation, repetition, or experiences of loss of control can be carefully and openly explored. The metasynthesis further indicates that many care strategies are already operative within chemsex settings themselves, expressed through tacit norms, informal accompaniment, and community-embedded harm reduction practices. Recognizing these existing resources does not amount to idealizing the practice; rather, it means identifying realistic entry points for more credible, co-produced, and less moralizing forms of intervention.

At the community and public health level, the results suggest that effective policies will be those capable of engaging with the ecological nature of chemsex: intervening at the individual level alone is inherently insufficient if the contexts that render the practice both accessible and desirable are left unaddressed. This includes, in particular, attending to the role of digital platforms in shaping opportunity structures and normative expectations, as well as to the ways in which certain sexual and community environments function simultaneously as spaces of recognition and as arenas of demand, performance, and hierarchy. Prevention efforts should accordingly move beyond the provision of information and instead prioritize accessible, non-stigmatizing care pathways—ones that attend specifically to mental health, sexuality, relational trauma, and substance use—while fostering articulated networks between clinical services, community resources, and culturally competent outreach strategies.

In sum, the aggregated qualitative evidence positions chemsex as a phenomenon in which pleasure, belonging, and vulnerability coexist in ways that are not easily disentangled. A proportionate response requires acknowledging this coexistence and designing interventions that do not compel individuals to choose between their health and their community but instead support the gradual reconstruction of forms of connection, desire, and emotional regulation that are less contingent on chemical enhancement and digital immediacy.

## Figures and Tables

**Table 1:** Search strategy.

**Topic of interest** Chemsex, addiction to sex and drugs, addiction, recreational drugs, sex and drugs, substance *, GHB, methamphetamine, poly-consumption, sex with partners, sex party, illicit drugs, sexualized drug use.
**Participants** Men, men who have sex with men, MSM, young men, adult men, men behavior, men psychology, men psychiatry, professionals, partners, teammates, mates, gay men, gay, bisexual, bisexual men.
**Qualitative** Qualitative research, interview, focus group, content analysis, nursing methodology, nursing research, metasynthesis, qualitative study, qualitative method, review, analytic review, literature review, systematic analysis, grounded theory, phenomenology, phenomenology interviews, ethnography.
**Variables** Perceptions, motiv *, definition *, knowledge, pleasure, self, technology, app, mobile app, mobile phone, attitudes, believes, self-concept, view, perspective, opinion, experience, culture, group, hedonism, subculture, experienced *, stigma, homophobia, internalized homophobia, self-stigma, low self-esteem, assertively, constructions, open to experience, personality, emotional process, emotion, regulation, strategies, coping, dilemma, implicative dilemma, values, subjective, motivation, cognitions, coping mechanism.

The asterisk is used as a truncation symbol to retrieve different word variants sharing the same root.

**Table 2:** Characteristics and main findings of the included studies.

Study	Location	Aim	Sample	Design	Key Findings
Ahmed et al. [12]	United Kingdom (South London)	To examine the nature and operation of social norms surrounding chemsex among gay men.	30 MSM interviewed (aged 21–53; 13 living with HIV) and 12 MSM in two focus groups.	Qualitative study; semi-structured interviews and focus groups; thematic analysis.	Chemsex was perceived as widespread and increasingly acceptable. Norms were organised around settings, permissions, expectations and personal boundaries; they could facilitate pleasure while also normalising potentially harmful practices.
Bourne et al. [3]	United Kingdom (South London)	To understand the personal and social contexts of chemsex, perceived harms and harm-reduction needs.	30 MSM aged 21–53; 13 living with HIV and 17 HIV-negative at their last test.	Exploratory qualitative study; semi-structured individual interviews; thematic analysis.	Drugs enhanced sex, but participants described overdose, difficulties negotiating consent, physical and mental health harms, and gaps in drug knowledge and specialist support. Fear of professional judgement complicated help-seeking.
Brooks-Gordon and Ebbitt [13]	United Kingdom	To investigate chemsex experiences and motivations in male sex work, with a focus on consent, economic exploitation and harm reduction.	14 healthcare providers and clients—including male sex workers and customers—aged 28–46.	Qualitative study; purposive sampling and semi-structured interviews; grounded-theory analysis.	The ‘consent ladder’ moved from willing, pleasurable and controlled participation towards blurred or broken consent, physical harm and financial exploitation. Re-empowerment strategies included peer support, integrated centres and smartphone-based support.
Dennermalm et al. [14]	Sweden/German (Stockholm–Berlin)	To understand drug use, sex on drugs and harm-reduction practices among Swedish MSM travelling to Berlin.	15 Swedish MSM aged 23–44.	Qualitative study; semi-structured interviews; thematic analysis.	Participants sought a workable balance between pleasure, safety and risk. Findings support non-judgemental, low-threshold services offering evidence-based information on dosing, overdose, long-term effects, injecting and HIV prevention.
Drysdale et al. [15]	Australia (four capital cities)	To examine the diversity and contextual variability of crystal methamphetamine use in sexual settings.	88 gay and bisexual men aged 21–74.	Qualitative interview study; grounded-theory analysis.	Drug-enhanced sex was practised across varied settings, relationships and patterns of use and was not inherently problematic or uniformly risky. Public-health responses should avoid treating the most harmful experiences as representative of all chemsex.
Eriksson [16]	Finland	To make chemsex visible in the Finnish context by analysing HIV-positive MSM’s experiences and perceptions.	17 gay men living with HIV, aged 36–72; 16 interviews (one with a couple).	Master’s thesis; semi-structured interviews; qualitative thematic analysis.	Participants recognised chemsex in Finland and used language that separated sexualised from non-sexual drug use. They identified both benefits and harms and called for sexologically competent, culturally sensitive treatment and stronger healthcare support beyond the third sector.
Flores-Aranda et al. [17]	Canada (Montréal)	To document current and former methamphetamine users’ chemsex experiences, patterns and service use, with particular attention to pleasure.	17 MSM: 9 current and 8 former methamphetamine users; aged 24–55.	Qualitative study; two focus groups; thematic analysis.	Pleasure involved sexual effects, relationships with self and others, and injecting. When pleasure diminished and harms accumulated, service use became more likely. Pleasure should therefore be addressed directly in chemsex care rather than treated as peripheral.
Graf et al. [18]	Germany (multiple cities)	To describe perceived prevalence, motivations and consequences of chemsex and the response of support services.	89 MSM across two qualitative projects, plus 27 professionals; MSM aged 22–64.	Qualitative synthesis of two interview studies; structured content analysis.	Men used drugs to intensify sensation and performance, reduce inhibition, increase intimacy and manage self-worth. Problematic use involved overdose, poorer wellbeing, unsafe sex and STIs; services should integrate sexual, psychological, social and substance-use support.
Hakim [19]	United Kingdom (London)	To interpret the emergence of chemsex in relation to its historical and material conditions.	15 gay and bisexual men aged 24–51, plus documentary material.	Qualitative cultural study; interviews and document analysis; conjunctural analysis.	Chemsex was interpreted as an embodied attempt to create collective intimacy under neoliberal conditions that privilege competitive individualism and weaken social bonds. This account challenges purely pathologising representations.
Hibbert et al. [20]	United Kingdom (Northern and Central England)	To understand MSM and service-provider perspectives on care for people engaging in chemsex or sexualised drug use.	13 MSM aged 23–66 and 16 service providers.	Qualitative study; semi-structured interviews; thematic analysis.	Sexual enhancement was a key motivation. Sexual-health services were generally valued, but access, insecure funding, provider attitudes and limited competence outside specialist services created barriers. Workforce training and sustained funding were recommended.
Jaspal [21]	United Kingdom (London and East Midlands)	To explore chemsex motivations and its functions in identity construction and coping with psychological stress.	16 gay and bisexual men aged 22–47; 6 living with HIV and 10 HIV-negative.	Qualitative interview study; thematic analysis informed by Identity Process Theory.	Homonegativity, rejection and HIV stigma threatened self-esteem and distinctiveness. Chemsex supported deflection-based coping through denial, self-concealment, transient depersonalisation, compartmentalisation and fantasy.
Joy et al. [22]	Canada (Vancouver, Ottawa and Halifax)	To examine how GBMSM use online technologies to negotiate sexualised drug use and sexual-health practices.	50 GBMSM aged 18–59.	Poststructural qualitative study; semi-structured interviews and brief survey; Foucauldian discourse analysis.	Participants negotiated sexualised drug use by contextualising digital and drug assemblages, constituting pleasure and practising ethical subjectivity. Their accounts framed it as chosen, relational and identity-forming—not solely as sexual-health risk.
Lim et al. [23]	Malaysia (Kuala Lumpur)	To understand motivations for, and management of, methamphetamine use in sexual settings among MSM.	20 MSM aged 21–43 (median 34) who used amphetamines for sexual purposes.	Qualitative study; semi-structured interviews; thematic analysis.	Methamphetamine increased libido, pleasure, stamina and disinhibition. Participants varied in their ability to control use and reduce harm, while stigma, criminalisation and limited culturally appropriate sexual-health and drug services impeded support.
Milhet et al. [24]	France (Paris)	To explore the dynamics of pleasure in individual chemsex experiences.	33 MSM aged 21–61; 17 living with HIV and 21 actively practising chemsex.	Qualitative interview study; content analysis.	Pleasure extended beyond bodily sensation to intimacy, love, sociability, sexual discovery and disinhibition. The same narratives also contained isolation, dependence, sexual assault and frustration, revealing the coexistence of pleasure and suffering.
Moyle et al. [25]	United Kingdom and online	To reframe sex–drug relations through the concept of pharmacosex and examine drug-enabled sexual enhancement beyond a risk-only lens.	45 interviewees across diverse gender and sexual identities, plus a virtual ethnography with 31 online contributors; aged 21–65.	Multi-study qualitative research; virtual ethnography and interviews; thematic and narrative analysis.	Drug use could intensify emotional connection, bodily sensation, desire and disinhibition and sometimes had therapeutic meaning. Shame, regret and harm coexisted with innovative practices of care, safety and risk management.
Nimbi et al. [26]	Italy	To investigate contexts, substance-use patterns, first chemsex experiences and harm-reduction attitudes.	30 MSM aged 26–62 (mean 39.7).	Qualitative study; semi-structured interviews; content analysis.	Chemsex was shaped by sociocultural context, privacy and stigma; some men travelled or used apps to preserve anonymity. Polysubstance use and varied pathways into chemsex were common, and views on formal harm-reduction initiatives were mixed.
Nimbi et al. [27]	Italy	To understand chemsex sexual experiences from a biopsychosocial and psychosexological perspective.	31 MSM aged 26–62 (mean 38.6).	Qualitative study; semi-structured interviews; thematic analysis.	Chemsex increased desire, arousal, disinhibition, duration, intimacy and pleasure but could impair erection or ejaculation. Motivations evolved from curiosity and social influence to coping and dependence; sober sex was often described as more intimate but more vulnerable to performance anxiety.
O’Byrne [28]	Canada	To explore why gay men use party drugs and how perceived benefits complicate risk-focused accounts.	17 gay men aged 26–46 who attended circuit parties, used party drugs and reported condomless sex.	Exploratory qualitative study; semi-structured interviews; thematic analysis informed by Deleuze and Bataille.	Party drugs were used to achieve desired sensations and to obtain respite from negative emotions. Benefits related to sex and sociality coexisted with risk, supporting non-stigmatising nursing care and pragmatic harm reduction.
Pires et al. [29]	Portugal (Lisbon)	To describe Lisbon’s chemsex scenes and the creation and early results of a community-led harm-reduction network.	No fixed sample: four authors’ professional experience, two discussion groups, participant observation and one practitioner/peer educator’s autoethnographic data.	Self-reflexive qualitative essay; participant observation, literature review, autoethnography and collective reflection.	Chemsex has locally specific forms that require tailored responses. Harm-reduction organisations can detect emerging needs, and community-led transdisciplinary networks can link drug, sexual-health, mental-health, diversity and violence services.
Pollard et al. [30]	United Kingdom (London and Brighton)	To explore marginalisation and the psychosocial context of chemsex among MSM.	15 gay men who practised chemsex, selected from a larger sample of 162; aged 20–44.	Qualitative telephone interviews; framework analysis informed by syndemic and minority-stress theories.	Chemsex was embedded in stigma, minority stress, risky environments and maladaptive coping. Themes included culture, intimacy and loneliness, reinforcing cycles, relationships, reducing or stopping use, and HIV/STI risk; interventions should address social context as well as behaviour.
Santoro et al. [31]	Spain (Madrid)	To identify and describe different types of chemsex sessions.	11 in-depth interview participants and 7 participants in two triangular focus groups; aged 22–46.	Qualitative study; interviews and triangular focus groups; thematic and sociological discourse analysis.	Four session types were identified: anonymous sessions, chill-sex, semi-closed parties among friends, and chemsex in saunas or other sex-on-premises venues. Their organisation, meanings and risks differed, so prevention should not treat chemsex as a single homogeneous practice.
Semple et al. [32]	United States (San Diego County)	To explore personal motivations for methamphetamine use and its interaction with risky sex among MSM living with HIV.	25 HIV-positive MSM aged 27–51.	Qualitative interview study; thematic analysis.	Methamphetamine was used for sexual enhancement and to self-medicate negative affect related to HIV status. Use was linked to multiple partners, sexual marathons, anonymous sex and low condom use; treatment should address both sexual motives and emotional distress.
Smith and Tasker [33]	United Kingdom (London)	To explore gay men’s narratives of chemsex and recovery following therapeutic intervention.	6 gay men aged 30–60.	Qualitative interview study; Labovian narrative analysis.	Narratives centred on acceptance and belonging, life near collapse and uncertainty about a future without chemsex. Chemsex was both identity-affirming and progressively isolating, explaining ambivalence about giving it up.
Souleymanov et al. [34]	Canada (Toronto)	To critically examine risk and pleasure discourses among gay and bisexual men who engage in party-and-play.	44 gay and bisexual men aged 20–69 (mean 37).	Qualitative study; one-hour in-depth interviews; critical discourse analysis.	Biomedical HIV discourse shaped participants’ perceptions and practices. Pleasure was mobilised as a language of self-control, while distinctions between acceptable and unacceptable pleasures reflected wider biopolitical regulation of sex and drugs.
Souleymanov et al. [35]	Canada (Toronto)	To examine social exclusion, resilience and social-worker preparedness in relation to online party-and-play.	44 gay and bisexual men who had used drugs before or during sex in the previous month; mean age 37.	Qualitative study; one-hour in-depth interviews; critical discourse analysis.	Participants described stigma, heteronormativity, homophobia, racism, classism and moralising risk discourses that restricted access to care. Resilience arose through social bonds and mutual care; social workers need culturally competent preparation.
Stanton et al. [36]	United States (online and community recruitment)	To explore how substance use promotes or hinders positive sexual expression and healthy relationships among MSM living with HIV.	33 MSM living with HIV, poorly engaged in care and reporting recent substance use; aged 26–68.	Secondary qualitative analysis of semi-structured interviews; thematic and content analysis informed by grounded theory.	Substances could enable intimacy, enhanced sex, access to partners or community, and sexual empowerment. These benefits sometimes came with disempowerment, unstable relationships and unequal power, underscoring the need to address relational motives in care.
Tan et al. [37]	Singapore	To investigate life histories of trauma underpinning sexualised drug use among treatment-experienced GBMSM.	33 purposively sampled GBMSM aged ≥21 with a history of sexualised drug use; most had treatment experience.	Qualitative study; semi-structured in-depth interviews; inductive thematic analysis.	Sexualised drug use generated pleasure, connection and relief but also functioned as coping. HIV stigma, racism, sexual violence, bereavement, neglect and internalised homophobia shaped trauma narratives; treatment should be trauma-informed and address structural barriers.
Tan et al. [38]	Singapore	To explore perceptions of and motivations for chemsex, and barriers to addressing associated harms.	30 GBMSM aged 18–39.	Qualitative descriptive study; semi-structured in-depth interviews; thematic analysis.	Motivations clustered around sexual enhancement, interpersonal pressures and broader social stressors. Punitive drug laws and criminalisation discouraged disclosure and help-seeking, limiting harm-reduction and culturally safe care.
Van Hout et al. [39]	Ireland (Dublin)	To explore sexualised drug-use pathways among help-seeking GBMSM experiencing physical or emotional consequences.	10 GBMSM aged 35–59.	Qualitative pilot study; semi-structured interviews; interpretative phenomenological analysis.	Themes covered social and digital arrangements, polydrug use and dependence, peer harm reduction, escapism and compulsive participation. Sexual pleasure and drug effects reinforced one another, while excess increasingly intersected with dependence.
Weatherburn et al. [40]	United Kingdom (South London)	To understand motivations and values associated with combining sex and illicit drugs.	30 gay men aged 21–53; 13 living with HIV.	Qualitative study; semi-structured interviews; thematic analysis with analytical triangulation.	Drugs enabled desired sex by increasing libido, confidence, disinhibition and stamina. They also intensified qualities men valued—attraction, sensation, intimacy and adventure—showing why risk-only messages may have limited resonance.
Wilkerson et al. [41]	United States (Texas)	To examine the association between chemsex and sexual violence and describe the contexts in which violence occurs.	Online survey of 1273 sexual and gender minority adults; separate qualitative sample of 22 substance-using sexual minority men aged 20–51.	Convergent mixed-methods study; logistic regression and semi-structured interviews with thematic analysis.	The chemsex proxy was associated with markedly higher odds of sexual violence. Interviews described intoxicated settings in which consent was difficult to revoke and exploitation was more likely, supporting clearer, context-specific approaches to consent and violence prevention.

Notes. MSM = men who have sex with men; GBMSM = gay, bisexual and other men who have sex with men; HIV = human immunodeficiency virus; STI = sexually transmitted infection. Ahmed et al. [12], Bourne et al. [3] and Weatherburn et al. [40] report distinct analyses of the same South London dataset. Pires et al. [29] did not use a conventional participant sample; its qualitative data sources are therefore reported instead.

**Table 3:** Contribution of Included Studies to the Six Interpretive Themes.

Study	Country/Setting	T1 (*n* = 10)	T2 (*n* = 30)	T3 (*n* = 26)	T4 (*n* = 29)	T5 (*n* = 25)	T6 (*n* = 31)
Ahmed et al. [12]	UK (South London)	●	●	●	●	●	●
Bourne et al. [3]	UK (South London)	●	●	●	●	●	●
Brooks-Gordon & Ebbitt [13]	UK		●		●	●	●
Dennermalm et al. [14]	Sweden/Germany		●		●	●	●
Drysdale et al. [15]	Australia		●	●	●	●	●
Eriksson [16]	Finland		●	●	●		●
Flores-Aranda et al. [17]	Canada (Québec)		●	●	●	●	●
Graf et al. [18]	Germany		●	●	●	●	●
Hakim [19]	UK (London)		●	●	●	●	●
Hibbert et al. [20]	England		●				●
Jaspal [21]	UK		●	●		●	●
Joy et al. [22]	Canada	●	●	●	●	●	●
Lim et al. [23]	Malaysia (Kuala Lumpur)		●	●	●		●
Milhet et al. [24]	France	●	●	●	●	●	●
Moyle et al. [25]	UK		●	●	●	●	●
Nimbi et al. [26]	Italy	●	●	●	●	●	●
Nimbi et al. [27]	Italy		●	●	●	●	●
O’Byrne [28]	Canada		●	●	●	●	●
Pires et al. [29]	Portugal (Lisbon)	●	●	●	●		●
Pollard et al. [30]	UK	●	●	●	●	●	●
Santoro et al. [31]	Spain (Madrid)	●	●	●	●	●	●
Semple et al. [32]	US (San Diego County)				●	●	●
Smith & Tasker [33]	UK (London)		●	●	●	●	●
Souleymanov et al. [34]	Canada (Toronto)		●	●	●	●	●
Souleymanov et al. [35]	Canada (Toronto)		●	●	●		●
Stanton et al. [36]	US (Boston)	●	●	●	●	●	●
Tan et al. [37]	Singapore		●	●	●	●	●
Tan et al. [38]	Singapore		●	●	●	●	●
Van Hout et al. [39]	Ireland (Dublin)	●	●	●	●	●	●
Weatherburn et al. [40]	UK (South London)		●	●	●	●	●
Wilkerson et al. [41]	US (Texas)		●		●		●
**Studies contributing, *n***		**10**	**30**	**26**	**29**	**25**	**31**

Note. ● indicates that the study supplied qualitative findings informing at least one code or subtheme within the corresponding interpretive theme; blank cells indicate that no direct contribution was identified in the extracted findings. T1 = Access to drugs and sex: “It is there when I want it”; T2 = Beliefs and attitudes toward chemsex: Internalising social constructions; T3 = Chemsex as an identity-based subculture; T4 = Patterns of use: Substances, relationships, and spaces; T5 = Motivations for chemsex: Pleasures and emotions; T6 = Self-perceived risks in chemsex: Beyond the biological. Themes were not mutually exclusive, so each study could contribute to more than one theme. Assignments were based on the qualitative findings reported in the included studies and were cross-checked against the metasynthesis coding structure, thematic results, and study summaries. Ahmed et al. [12], Bourne et al. [3], and Weatherburn et al. [40] analysed the same South London dataset but addressed distinct analytic questions. UK = United Kingdom; US = United States.

## Data Availability

No new data were created or analyzed in this study. Data sharing is not applicable to this article.

## Supplementary Materials

The following supporting information can be downloaded at: https://www.mdpi.com/article/10.3390/healthcare14183071/s1, Table S1: Enhancing Transparency in Reporting the Synthesis of Qualitative Research (ENTREQ) Checklist; Table S2: PRISMA 2020 Checklist; Table S3: CASPe scores of the studies included in the critical appraisal stage.
