# Supplementary material for: Experiences, Beliefs, and Dynamics in Chemsex: A Qualitative Meta-Synthesis Among Gay, Bisexual, and Other Men Who Have Sex with Men
Source: Healthcare (Basel). 2026 Sep 18;14(18):3071. doi: 10.3390/healthcare14183071 (PMC13606892; doi:10.3390/healthcare14183071)
Supplement: Supplementary file 1 [file healthcare-14-03071-s001.zip › healthcare-4332180-supplementary.pdf]

Supplementary Materials

Experiences, Beliefs, and Dynamics in Chemsex: A Qualitative Meta-Synthesis on Sexualized Drug Use among Gay, Bisexual, and Other Men Who Have Sex with Men

Manuscript ID: healthcare-4332180

Table S1. Enhancing Transparency in Reporting the Synthesis of Qualitative Research (ENTREQ) Checklist

Item No.	Guide and Description	Report Location in the Current Manuscript
1	Aim. State the research question the synthesis addresses.	Reported. Abstract, “Objective”; Introduction, final paragraph; and Materials and Methods, opening paragraph.
2	Synthesis methodology. Identify the synthesis methodology or theoretical framework underpinning the synthesis and describe the rationale for its selection (e.g., meta-ethnography, thematic synthesis, critical interpretive synthesis, grounded theory synthesis, realist synthesis, meta-aggregation, meta-study, or framework synthesis).	Reported. Materials and Methods, opening paragraph and final synthesis paragraph. The manuscript identifies a qualitative meta-synthesis using inductive thematic analysis and explains that the purpose was interpretive integration beyond aggregation, progressing from descriptive coding to higher-order analytical themes.
3	Approach to searching. Indicate whether the search was pre-planned (a comprehensive strategy seeking all available studies) or iterative (seeking concepts until theoretical saturation is achieved).	Partially reported. Materials and Methods, database-search paragraph, describes a systematic search; Table 1 presents predefined search domains. The manuscript does not explicitly label the approach as “pre-planned” or “iterative,” nor does it discuss theoretical saturation.
4	Inclusion criteria. Specify the inclusion and exclusion criteria (e.g., population, language, year limits, type of publication, and study type).	Reported. Materials and Methods, database-search and eligibility/critical-appraisal paragraphs. Population, study designs, qualitative-content requirement, publication languages, absence of time restrictions, and exclusion criteria are stated.
5	Data sources. Describe the information sources used (e.g., electronic databases, grey literature, organisational websites, experts, generic web searches, hand searching, and reference lists), when the searches were conducted, and the rationale for using them.	Rreported. Materials and Methods, database-search and grey-literature paragraphs: PubMed, Scopus, CINAHL, PubPsych, Dialnet, and a purposive review of grey literature. The rationale for Dialnet and for using no date restriction is stated.
6	Electronic search strategy. Describe the literature search, including population terms, health-topic terms, experiential or social-phenomenon terms, qualitative-research filters, and search limits.	Reported. Materials and Methods, database-search paragraph, and Table 1, “Searching strategy.” The manuscript provides four term domains (topic, participants, qualitative methods, and variables) and states the language and time limits.
7	Study screening methods. Describe the study-screening and sifting process (e.g., title, abstract, and full-text review, and the number of independent reviewers who screened studies).	Reported. Materials and Methods, selection-process paragraph, and Table 2, “Methodological strategy of the meta-synthesis.” Title/abstract screening, full-text assessment, and recording of exclusion reasons are described.
8	Study characteristics. Present the characteristics of the included studies (e.g., publication year, country, population, participant number, data collection, methodology, analysis, and research questions).	Reported. Results, opening paragraph, provides aggregate characteristics for the 31 studies (publication period, geographical distribution, settings, data-collection methods, and analytical approaches).
9	Study selection results. Identify the number of studies screened and provide reasons for exclusion. For a comprehensive search, report screening numbers and exclusion reasons in a figure or flowchart.	Reported. Abstract, “Results,” and Table 2 provide identification, screening, eligibility, exclusion-reason, and inclusion counts.

Item No.	Guide and Description	Report Location in the Current Manuscript
10	Rationale for appraisal. Describe the rationale and approach used to appraise the included studies or selected findings (e.g., conduct and robustness, reporting transparency, or content and utility of findings).	Reported. Materials and Methods, eligibility/critical-appraisal paragraph. Appraisal is described as a reasoned judgement of methodological rigor rather than a simple score, intended to ensure a minimum level of methodological consistency.
11	Appraisal items. State the tools, frameworks, and criteria used to appraise the studies or selected findings (e.g., CASP, QARI, COREQ, or reviewer-developed tools, and the domains assessed).	Reported. Abstract, “Methods,” and Materials and Methods, eligibility/critical-appraisal paragraph, identify the Critical Appraisal Skills Programme (CASPe) tool.
12	Appraisal process. Indicate whether appraisal was conducted independently by more than one reviewer and whether consensus was required.	Reported and Methods states that critical appraisal was performed and identify the appraisers, independence, consensus procedures, or adjudication.
13	Appraisal results. Present the quality-assessment results and indicate which articles, if any, were weighted or excluded on this basis, with the rationale.	Reported. The manuscript present the quality-assessment results.
14	Data extraction. Indicate which sections of the primary studies were analysed and how data were extracted from the primary studies.	Not reported in the current manuscript. No section specifies which portions of the primary reports constituted data, what information was extracted, who extracted it, or how extraction was recorded and checked.
15	Software. State the computer software used, if any.	Not reported in the current manuscript. The article should name the software used or explicitly state that no dedicated qualitative-analysis software was used.
16	Number of reviewers. Identify who was involved in coding and analysis.	Not reported in the current manuscript. The Acknowledgments mention collaborators in screening, appraisal, and discussion, but the number, identity, and roles of those who coded and analysed the data are not specified.
17	Coding. Describe the process used to code the data (e.g., line-by-line coding to identify concepts).	Reported at a general level. Materials and Methods, final synthesis paragraph, describes progressive inductive coding, development of descriptive themes, grouping, reinterpretation, and construction of higher-order analytical themes. Results, opening paragraph, reports 276 codes. The coding unit and operational detail (e.g., line-by-line coding) are not specified.
18	Study comparison. Describe how comparisons were made within and across studies (e.g., coding later studies into existing concepts and creating new concepts when necessary).	Reported at a general level. Materials and Methods, final synthesis paragraph, states that convergences and tensions were integrated across studies while contextual differences and nuances were retained. The sequential procedure for constant comparison is not described in detail.
19	Derivation of themes. Explain whether the themes or constructs were derived inductively or deductively.	Reported. Abstract, “Methods”; Materials and Methods, final synthesis paragraph; and Results, opening paragraph, explicitly describe an inductive thematic process in which 276 codes were reorganized into six interpretive themes.

Item No.	Guide and Description	Report Location in the Current Manuscript
20	Quotations. Provide quotations from the primary studies to illustrate themes or constructs, and identify whether they are participant quotations or authors’ interpretations.	Partially reported. Results, all six thematic subsections, includes illustrative quotations with citations to their primary-study sources. The extracts are presented as participant accounts, but the manuscript does not consistently label the distinction between first-order participant quotations and primary authors’ interpretations.
21	Synthesis output. Present rich, compelling, and useful results that go beyond a summary of the primary studies (e.g., new interpretations, conceptual models, analytical frameworks, or development of theory or constructs).	Reported. Results, Discussion, and Conclusions develop an interpretive account of chemsex as a relational ecology shaped by digital mediation, subcultural belonging, shared meanings, ambivalence, and cumulative harms, and derive clinical and public-health implications beyond descriptive aggregation.

Note. *Adapted from Tong et al. [7].*

Table S2. PRISMA 2020 Checklist

Section and topic	Item	Checklist item	Location where item is reported
**TITLE**	**TITLE**	**TITLE**	**TITLE**
Title	1	Identify the report as a systematic review.	Partially reported. Title (p. 1). The title identifies the report as a "qualitative meta-synthesis," but does not explicitly use the term "systematic review."
**ABSTRACT**	**ABSTRACT**	**ABSTRACT**	**ABSTRACT**
Abstract	2	See the PRISMA 2020 for Abstracts checklist.	Reported. Abstract (p. 1). The structured abstract reports the background, objective, information sources, broad eligibility criteria, critical appraisal method, synthesis method, selection counts, principal themes, conclusion, and implications.
**INTRODUCTION**	**INTRODUCTION**	**INTRODUCTION**	**INTRODUCTION**
Rationale	3	Describe the rationale for the review in the context of existing knowledge.	Reported. Introduction (pp. 2–3), particularly the final two paragraphs. The manuscript explains that qualitative evidence is dispersed across fields and theoretical approaches and that an interpretive synthesis is needed to integrate experiential, social, and relational dimensions.
Objectives	4	Provide an explicit statement of the objective(s) or question(s) the review addresses.	Reported. Abstract, "Objective" (p. 1); Introduction, final paragraph (p. 3); and Materials and Methods, opening paragraph (p. 3).
**METHODS**	**METHODS**	**METHODS**	**METHODS**
Eligibility criteria	5	Specify the inclusion and exclusion criteria for the review and how studies were grouped for the syntheses.	Reported. Materials and Methods (p. 3). The manuscript specifies the population, eligible study designs, qualitative-content requirement, languages, absence of time restrictions, exclusion criteria, and handling of reviews to prevent double counting. All eligible studies entered the qualitative synthesis; their contributions to the six themes are mapped in Table 4 (pp. 23–24).
Information sources	6	Specify all databases, registers, websites, organisations, reference lists and other sources searched or consulted to identify studies. Specify the date when each source was last searched or consulted.	Reported. Materials and Methods (p. 3). PubMed, Scopus, CINAHL, PubPsych, Dialnet, and a purposive review of grey literature are reported, and the overall search period is October 2022–January 2023.
Search strategy	7	Present the full search strategies for all databases, registers and websites, including any filters and limits used.	Reported. Materials and Methods (p. 3) and Table 1, "Searching strategy" (p. 4). The manuscript reports four search-term domains, Boolean logic, languages, and the absence of date restrictions.

Section and topic	Item	Checklist item	Location where item is reported
Selection process	8	Specify the methods used to decide whether a study met the inclusion criteria of the review, including how many reviewers screened each record and each report retrieved, whether they worked independently, and if applicable, details of automation tools used in the process.	Reported. Materials and Methods (pp. 3–4). Selection by three pairs of reviewers and a pair of independent judges is stated, and title/abstract and full-text phases are described.
Data collection process	9	Specify the methods used to collect data from reports, including how many reviewers collected data from each report, whether they worked independently, any processes for obtaining or confirming data from study investigators, and if applicable, details of automation tools used in the process.	Partially reported. Materials and Methods (pp. 3 and 5). The manuscript explains how primary qualitative evidence was distinguished from secondary interpretations in eligible reviews and how Spanish-language material was extracted, coded, translated, and back-translated. It does not fully describe the extraction procedure for all reports, the number and independence of extractors, checking or author-contact procedures, the extraction form, or any automation.
Data items	10a	List and define all outcomes for which data were sought. Specify whether all results that were compatible with each outcome domain in each study were sought (e.g. for all measures, time points, analyses), and if not, the methods used to decide which results to collect.	Not applicable to quantitative outcomes. This is a qualitative meta-synthesis and no numerical outcome measures were sought. The experiential and sociocultural phenomena of interest are stated in the objective (pp. 1 and 3) and in the “Variables” domain of Table 1 (p. 4), but the manuscript does not explicitly state whether all eligible qualitative findings from each report were extracted.
Data items	10b	List and define all other variables for which data were sought (e.g. participant and intervention characteristics, funding sources). Describe any assumptions made about any missing or unclear information.	Reported. Table 3, “Characteristics and main findings of the included studies” (pp. 17–22), presents author/year, location, aim, sample, design, and key findings.
Study risk of bias assessment	11	Specify the methods used to assess risk of bias in the included studies, including details of the tool(s) used, how many reviewers assessed each study and whether they worked independently, and if applicable, details of automation tools used in the process.	Reported. Abstract (p. 1) and Materials and Methods (p. 3). CASPe was used as a minimum methodological-rigor threshold rather than to weight findings.
Effect measures	12	Specify for each outcome the effect measure(s) (e.g. risk ratio, mean difference) used in the synthesis or presentation of results.	Not applicable. The review synthesizes qualitative findings and does not calculate quantitative effect measures.
Synthesis methods	13a	Describe the processes used to decide which studies were eligible for each synthesis (e.g. tabulating the study intervention characteristics and comparing against the planned groups for each synthesis (item #5)).	Reported. Eligibility criteria are described in Materials and Methods (p. 3). All 31 included studies entered the qualitative meta-synthesis, and Table 4 (pp. 23–24) maps which studies contributed to each of the six interpretive themes.
Synthesis methods	13b	Describe any methods required to prepare the data for presentation or synthesis, such as handling of missing summary statistics, or data conversions.	Partially reported. Materials and Methods (pp. 3 and 5). The manuscript describes avoiding double counting when extracting primary evidence from reviews and describes original-language coding, English translation, and back-translation for the Spanish-language study. No general procedure for preparing, standardizing, or managing missing/unclear qualitative data is reported.

Section and topic	Item	Checklist item	Location where item is reported
Synthesis methods	13c	Describe any methods used to tabulate or visually display results of individual studies and syntheses.	Reported. Results and Supplementary Material use a study-characteristics table (Table 3, pp. 17–22) and a study-by-theme contribution matrix (Table 4, pp. 23–24).
Synthesis methods	13d	Describe any methods used to synthesize results and provide a rationale for the choice(s). If meta-analysis was performed, describe the model(s), method(s) to identify the presence and extent of statistical heterogeneity, and software package(s) used.	Reported. Materials and Methods (pp. 3 and 5). The manuscript justifies interpretive integration beyond aggregation and describes inductive thematic analysis progressing from codes and descriptive themes to higher-order analytical themes, using ATLAS.ti. No meta-analysis was performed.
Synthesis methods	13e	Describe any methods used to explore possible causes of heterogeneity among study results (e.g. subgroup analysis, meta-regression).	Reported for a qualitative synthesis. Materials and Methods (p. 5) states that convergences and tensions were integrated while contextual differences and nuances were retained.
Synthesis methods	13f	Describe any sensitivity analyses conducted to assess robustness of the synthesized results.	Not reported. The manuscript does not describe a sensitivity analysis or explicitly state that none was conducted.
Reporting bias assessment	14	Describe any methods used to assess risk of bias due to missing results in a synthesis (arising from reporting biases).	Reported. A method for assessing bias due to missing qualitative findings or selective reporting is described.
Certainty assessment	15	Describe any methods used to assess certainty (or confidence) in the body of evidence for an outcome.	Not reported. No formal assessment of confidence in the synthesized qualitative findings (e.g., GRADE-CERQual) is described.
**RESULTS**	**RESULTS**	**RESULTS**	**RESULTS**
Study selection	16a	Describe the results of the search and selection process, from the number of records identified in the search to the number of studies included in the review, ideally using a flow diagram.	Reported. The results show the search, selection process, number of records and number of studies included.
Study selection	16b	Cite studies that might appear to meet the inclusion criteria, but which were excluded, and explain why they were excluded.	Not reported. Table 2 (pp. 4–5) gives aggregate exclusion reasons, but potentially eligible excluded reports are not individually cited with reasons.
Study characteristics	17	Cite each included study and present its characteristics.	Reported. Results, opening paragraph (p. 6), and Table 3, “Characteristics and main findings of the included studies” (pp. 17–22). Each of the 31 included studies is cited and summarized by location, aim, sample, design, and key findings.
Risk of bias in studies	18	Present assessments of risk of bias for each included study.	Not reported in the current manuscript file. Materials and Methods (p. 3) states that a detailed study-level CASPe appraisal is available in Supplementary Material, but no such table appears in the supplied file.

Section and topic	Item	Checklist item	Location where item is reported
Results of individual studies	19	For all outcomes, present, for each study: (a) summary statistics for each group (where appropriate) and (b) an effect estimate and its precision (e.g. confidence/credible interval), ideally using structured tables or plots.	Reported in the form applicable to a qualitative synthesis. Table 3 (pp. 17–22) presents the main qualitative findings for each included study. Group summary statistics, effect estimates, and precision measures are not applicable because no quantitative outcomes or meta-analysis were undertaken.
Results of syntheses	20a	For each synthesis, briefly summarise the characteristics and risk of bias among contributing studies.	Partially reported. Results, opening paragraph (p. 6), summarizes publication years, geographical settings, data-collection methods, and analytic approaches; Table 4 (pp. 23–24) maps study contributions to each theme. Study-level risk-of-bias results are absent from the current file.
Results of syntheses	20b	Present results of all statistical syntheses conducted. If meta-analysis was done, present for each the summary estimate and its precision (e.g. confidence/credible interval) and measures of statistical heterogeneity. If comparing groups, describe the direction of the effect.	Not applicable. No statistical synthesis or meta-analysis was conducted.
Results of syntheses	20c	Present results of all investigations of possible causes of heterogeneity among study results.	Not applicable to a formal statistical investigation. Contextual and methodological variation is interpreted narratively in the Results (pp. 6–10), Discussion (pp. 10–12), and Limitations (p. 12), but no formal heterogeneity analysis was conducted.
Results of syntheses	20d	Present results of all sensitivity analyses conducted to assess the robustness of the synthesized results.	Not reported. No sensitivity analysis is described or presented.
Reporting biases	21	Present assessments of risk of bias due to missing results (arising from reporting biases) for each synthesis assessed.	Not reported. No assessment of reporting bias or missing qualitative findings is presented.
Certainty of evidence	22	Present assessments of certainty (or confidence) in the body of evidence for each outcome assessed.	Not reported. No formal confidence assessment for the six qualitative themes is presented.
**DISCUSSION**	**DISCUSSION**	**DISCUSSION**	**DISCUSSION**
Discussion	23a	Provide a general interpretation of the results in the context of other evidence.	Reported. Discussion (pp. 10–12) and Conclusions (pp. 12–14) interpret the six themes in relation to external evidence and develop chemsex as a relational and situated phenomenon shaped by digital mediation, subcultural belonging, ambivalence, and risk.
Discussion	23b	Discuss any limitations of the evidence included in the review.	Reported. “Limitations” (p. 12). The manuscript discusses geographical and service-connected sampling concentration, underrepresented experiences, conceptual and methodological heterogeneity, retrospective self-report, stigma, and uneven reporting in primary studies.

Section and topic	Item	Checklist item	Location where item is reported
Discussion	23c	Discuss any limitations of the review processes used.	Reported. “Limitations” (p. 12). The manuscript acknowledges the interpretive dependence of coding, thematic aggregation, and integration, the impossibility of fully standardizing these decisions, and the influence of uneven detail in primary reports.
Discussion	23d	Discuss implications of the results for practice, policy, and future research.	Reported. Discussion (pp. 10–12) and Conclusions (pp. 12–14) provide detailed clinical, harm-reduction, community, and public-health implications.
**OTHER INFORMATION**	**OTHER INFORMATION**	**OTHER INFORMATION**	**OTHER INFORMATION**
Registration and protocol	24a	Provide registration information for the review, including register name and registration number, or state that the review was not registered.	Reported. Materials and Methods (p. 3): PROSPERO registration CRD42023434687 (Record ID/CRD Accession Number 434687).
Registration and protocol	24b	Indicate where the review protocol can be accessed, or state that a protocol was not prepared.	Reported through the registry identification. Materials and Methods (p. 3) identifies the PROSPERO record and registration number, enabling access to the registered protocol; no direct URL is provided.
Registration and protocol	24c	Describe and explain any amendments to information provided at registration or in the protocol.	Not reported. The manuscript does not state whether the registered protocol was amended or whether there were no amendments.
Support	25	Describe sources of financial or non-financial support for the review, and the role of the funders or sponsors in the review.	Reported. Funding statement (p. 14): “This research received no external funding.” No funder or sponsor role therefore applies.
Competing interests	26	Declare any competing interests of review authors.	Reported. Conflicts of Interest statement (p. 14): the authors declare no conflict of interest.
Availability of data, code and other materials	27	Report which of the following are publicly available and where they can be found: template data collection forms; data extracted from included studies; data used for all analyses; analytic code; any other materials used in the review.	Reported. Data Availability Statement (p. 14) states that no new data were created and that supporting data are available within the article and the cited primary studies. The availability of extraction forms, the complete extracted dataset, the ATLAS.ti project/coding structure, analytic code, and other review materials is specified.

Note. *Adapted from Page et al. [8].*

Table S3. CASPe Scores of the Studies Included in the Critical Appraisal Stage

Study	CASPe score	Included/ Excluded
Ahmed et al. [12]	8	Included
Bourne et al. [3]	8	Included
Brooks-Gordon and Ebbitt [13]	10	Included
Dennermalm et al. [14]	9	Included
Drysdale et al. [15]	8	Included
Eriksson [16]	9	Included
Flores-Aranda et al. [17]	8	Included
Graf et al. [18]	9	Included
Hakim [19]	10	Included
Hibbert et al. [20]	9	Included
Jaspal [21]	8	Included
Joy et al. [22]	10	Included
Lim et al. [23]	9	Included
Milhet et al. [24]	8	Included
Moyle et al. [25]	9	Included
Nimbi et al. [26]	9	Included
Nimbi et al. [27]	9	Included
O'Byrne [28]	9	Included
Pires et al. [29]	9	Included
Pollard et al. [30]	9	Included
Santoro et al. [31]	10	Included
Semple et al. [32]	8	Included
Smith and Tasker [33]	9	Included
Souleymanov et al. [34]	9	Included
Souleymanov et al. [35]	9	Included

Study	CASPe score	Included/ Excluded
Stanton et al. [36]	9	Included
Tan et al. [37]	10	Included
Tan et al. [38]	9	Included
Van Hout et al. [39]	9	Included
Weatherburn et al. [40]	8	Included
Wilkerson et al. [41]	9	Included
Evans [53]	4	Excluded
O’Reilly [54]	4	Excluded

Note. *Each affirmative response (“Yes”) was assigned 1 point; “No” and “Can’t tell” were assigned 0 points. CASPe = Critical Appraisal Skills Programme.*
